# Supplementary material for: Analysis of the Whole-Genome Sequences from an Equus Parent-Offspring Trio Provides Insight into the Genomic Incompatibilities in the Hybrid Mule
Source: Genes (Basel). 2022 Nov 23;13(12):2188. doi: 10.3390/genes13122188 (PMC9778318; doi:10.3390/genes13122188)
Supplement: Supplementary file 1 [file genes-13-02188-s001.zip › genes-1972708-SI/Supplemental material.pdf]

## Supplemental material:

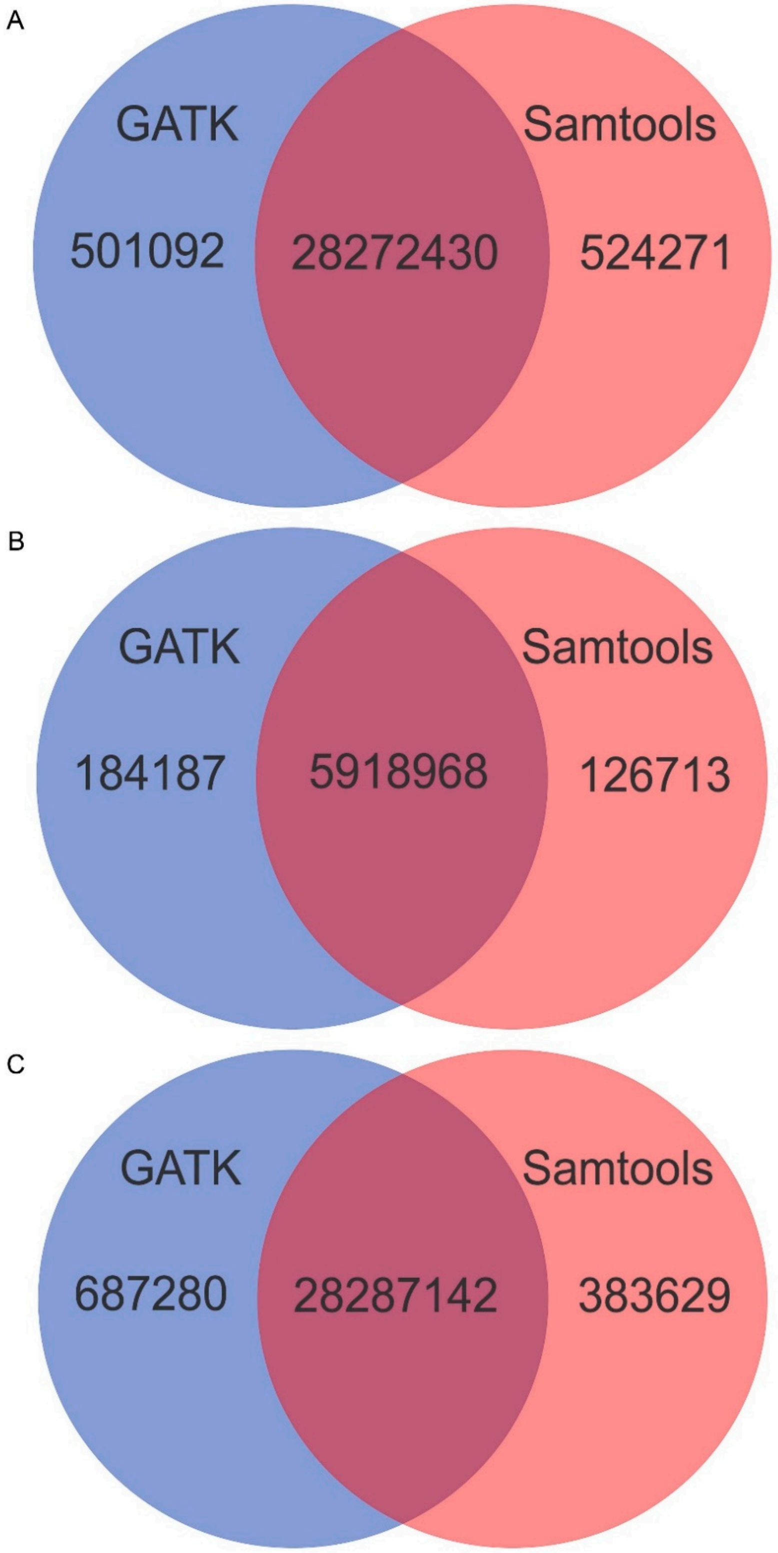


**Figure S1. Venn diagram showing unique and shared SNPs identified by two software in (A) donkey, (B) horse, and (C) mule genomes. SAMtools and GATK were used to call SNPs for each animal. The number of SNPs is listed in each diagram component.**

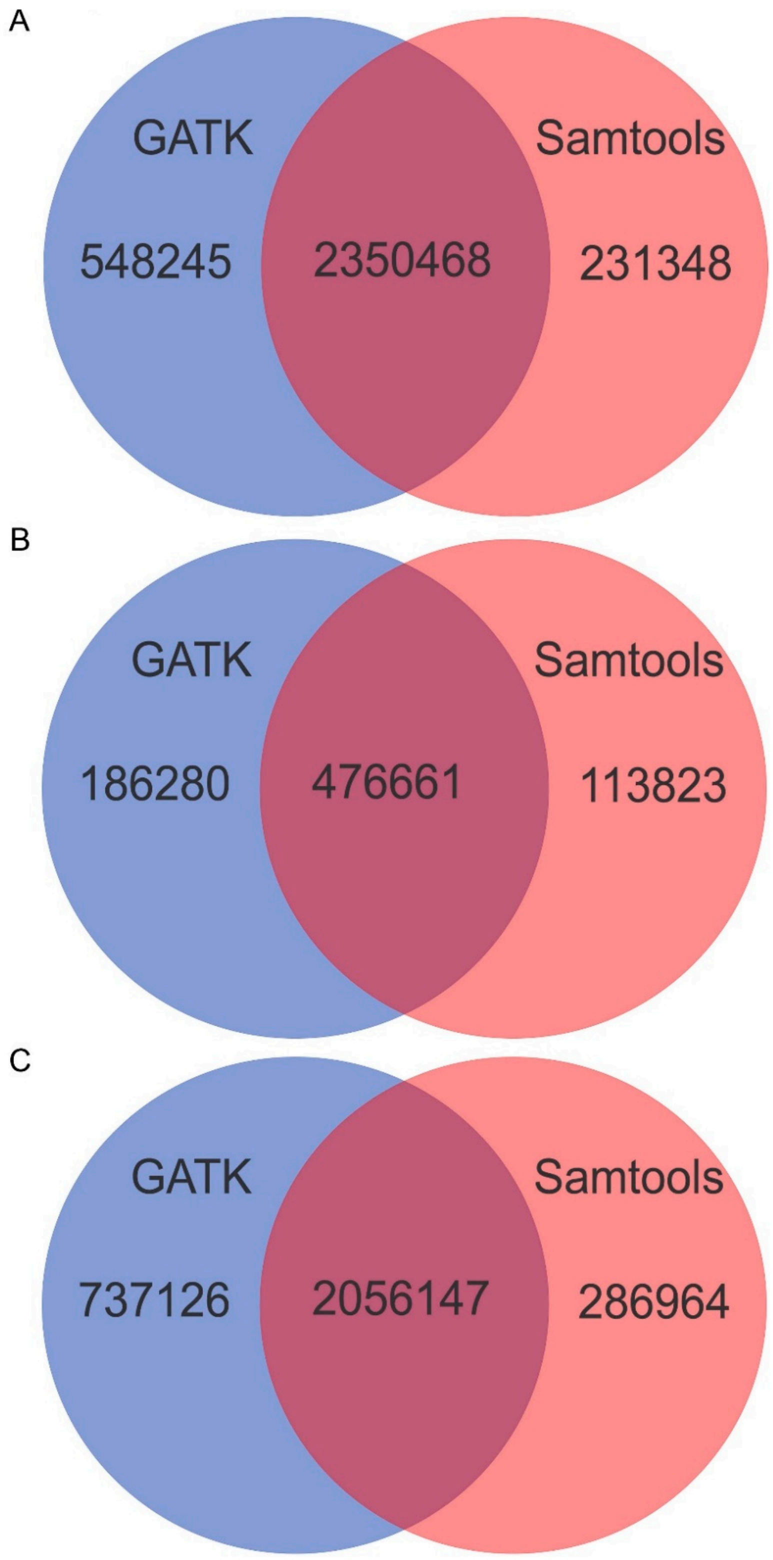


**Figure S2. Venn diagram showing unique and shared InDels identified by two software in (A) donkey, (B) horse, and (C) mule genomes. SAMtools and GATK were used to call InDels for each animal. The number of SNPs is listed in each diagram component.**

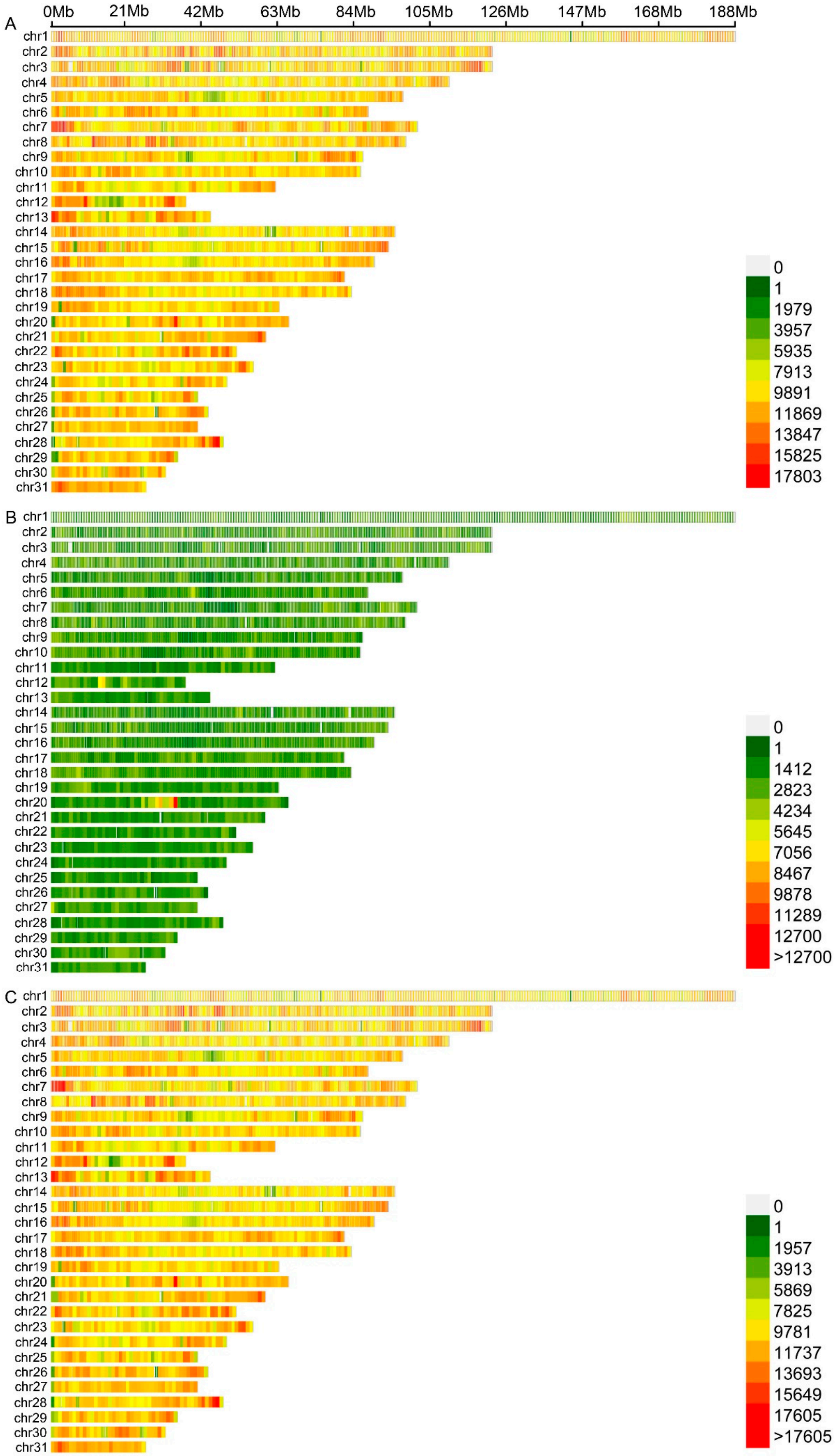
A
B
C
0Mb
21Mb
42Mb
63Mb
84Mb
105Mb
126Mb
147Mb
168Mb
188Mb
chr1
chr2
chr3
chr4
chr5
chr6
chr7
chr8
chr9
chr10
chr11
chr12
chr13
chr14
chr15
chr16
chr17
chr18
chr19
chr20
chr21
chr22
chr23
chr24
chr25
chr26
chr27
chr28
chr29
chr30
chr31
0
1
1979
3957
5935
7913
9891
11869
13847
15825
17803
0
1
1412
2823
4234
5645
7056
8467
9878
11289
12700
>12700
0
1
1957
3913
5869
7825
9781
11737
13693
15649
17605
>17605

**Figure S3. The distribution of SNPs on autosome for (A) the donkey, (B) the horse and (C) the mule. The distribution was based on values calculated for each sliding window of 1 Mb. SNPs are evenly distributed on the chromosomes for the donkey, the horse and the mule.**

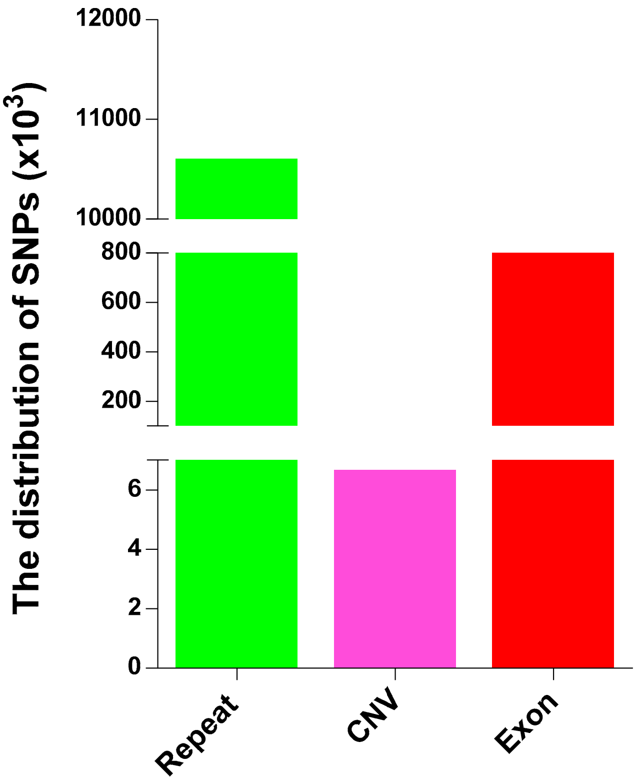


**Figure S4. The annotation of SNPs. RepeatMasker was used to define the repetitive elements. CNVnator was used to identify the Copy number variation sequences. The gtf annotation file defined the exonic regions in the reference genome. Repetitive elements and CNV sequences affect the accuracy of assigning alleles to their parental genomic inheritance. SNPs masked out repeats and CNVs were used for subsequent analysis.**

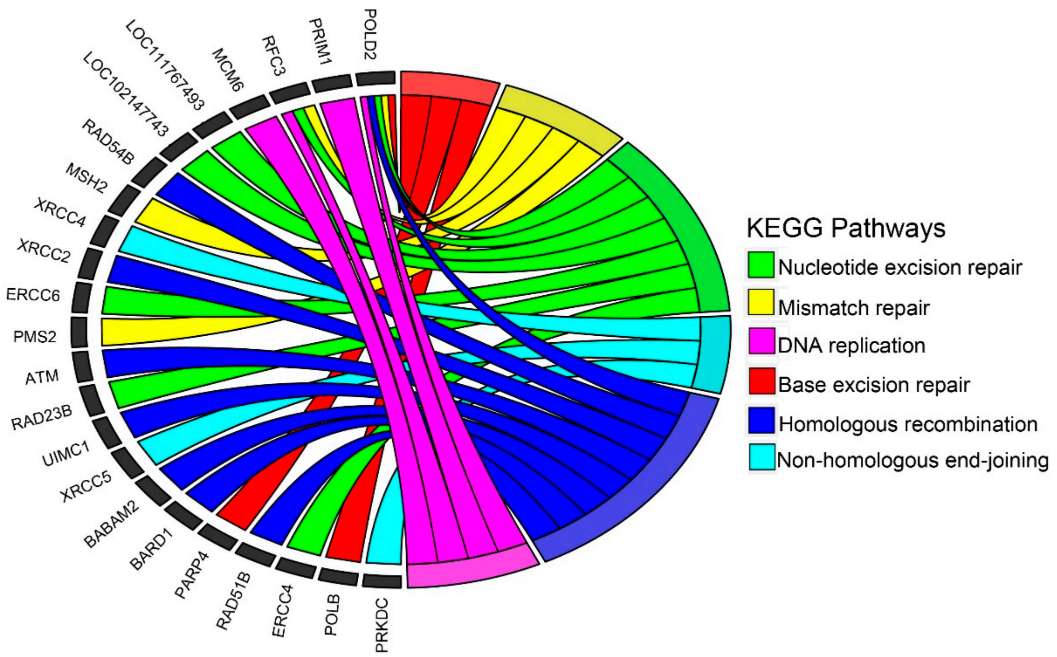


**Figure S5. Summary of point mutation genes involved in DNA repair processes in the mule genome.**

**Table S1. Statistics regarding mapping of donkey raw reads to the Thoroughbred horse genome (*EquCab3.0*)**

| Chr. | Total bases | Mapped base | no depth filter | ≥5 depth | coverage (no depth filter) | coverage (up to 5 depth) |
|---|---|---|---|---|---|---|
| 1 | 188260577 | 6933738386 | 186906088 | 185656122 | 99.28052436 | 98.61657 |
| 2 | 121350024 | 3975018757 | 120429542 | 119658530 | 99.24146533 | 98.6061 |
| 3 | 121351753 | 4016612392 | 120729797 | 119881486 | 99.48747671 | 98.78843 |
| 4 | 109462549 | 3499907052 | 108636268 | 107969427 | 99.24514731 | 98.63595 |
| 5 | 96759418 | 3178257163 | 95984059 | 95444462 | 99.19867335 | 98.641 |
| 6 | 87230776 | 2809940268 | 86652506 | 85976297 | 99.3370803 | 98.56188 |
| 7 | 100787686 | 3294563337 | 99933386 | 99062557 | 99.15237661 | 98.28835 |
| 8 | 97563019 | 3350305724 | 96058038 | 95260712 | 98.45742678 | 97.64018 |
| 9 | 85793548 | 2735920269 | 84778716 | 84082383 | 98.81712317 | 98.00549 |
| 10 | 85155674 | 3097260609 | 84389632 | 83736463 | 99.10042166 | 98.33339 |
| 11 | 61676917 | 1971667560 | 61244278 | 60815736 | 99.29853984 | 98.60372 |
| 12 | 36992759 | 1362857439 | 35949218 | 35104286 | 97.17906685 | 94.89502 |
| 13 | 43784481 | 1549646634 | 43155086 | 42638936 | 98.56251579 | 97.38367 |
| 14 | 94600235 | 3003564646 | 94071199 | 93436691 | 99.44076672 | 98.77004 |
| 15 | 92851403 | 2996733291 | 92227422 | 91582854 | 99.32797892 | 98.63379 |
| 16 | 88962352 | 2883668661 | 88610657 | 88222692 | 99.60466985 | 99.16857 |
| 17 | 80722430 | 2573459192 | 80349123 | 80051418 | 99.53754242 | 99.16874 |
| 18 | 82641348 | 2689757940 | 82220237 | 81839730 | 99.49043547 | 99.03 |
| 19 | 62681739 | 2006104571 | 61838376 | 61445502 | 98.65453159 | 98.02776 |
| 20 | 65343332 | 2208315840 | 64404978 | 63833033 | 98.56396365 | 97.68867 |
| 21 | 58984458 | 1879936250 | 58580814 | 58199270 | 99.31567736 | 98.66882 |
| 22 | 50928189 | 1622538896 | 50612940 | 50230091 | 99.38099311 | 98.62925 |
| 23 | 55556184 | 1797486921 | 55128985 | 54718964 | 99.2310505 | 98.49302 |
| 24 | 48288683 | 1522389498 | 47709820 | 47167938 | 98.801245 | 97.67907 |
| 25 | 40282968 | 1291714357 | 39759144 | 39398579 | 98.69963901 | 97.80456 |
| 26 | 43147642 | 2389062794 | 42525066 | 42112744 | 98.55710307 | 97.6015 |
| 27 | 40254690 | 1268154216 | 39476980 | 39166321 | 98.06802636 | 97.29629 |
| 28 | 47348498 | 1558671734 | 46534706 | 46176768 | 98.28127177 | 97.52531 |
| 29 | 34776120 | 1080511150 | 33607725 | 33066980 | 96.64023761 | 95.08531 |
| 30 | 31395959 | 947476205 | 30977459 | 30725747 | 98.66702591 | 97.86529 |
| 31 | 26001039 | 2071946985 | 25893137 | 25774990 | 99.58500889 | 99.13062 |
| Avg. | - | - | - | - | 98.90984 | 98.10537 |

**Table S2. Summary of genome sequencing for the male donkey.**

| Library type | Number of library | Insert size(bp) | Platform | Reads length (bp) | Raw data (Gb) | clean data (Gb) |
|---|---|---|---|---|---|---|
| **PE400** | 1 | 400 | MiSeq | Paired-End, 2×251 | 13.12 | 10.32 |
| **PE450** | 3 | 450 | MiSeq | Paired-End, 2×251 | 31.74 | 21.73 |
| **PE700** | 3 | 700 | MiSeq | Paired-End, 2×251 | 25.04 | 16.25 |
| **PE1000** | 1 | 1000 | MiSeq | Paired-End, 2×251 | 3.97 | 1.54 |
| **MP3K** | 2 | 3000 | HiSeq2000 | Mate-Paired, 2×100 | 27.80 | 15.60 |
| **MP5K** | 2 | 5000 | HiSeq2000 | Mate-Paired, 2×100 | 24.40 | 14.26 |
| **MP8K** | 2 | 8000 | HiSeq2000 | Mate-Paired, 2×100 | 23.42 | 13.18 |
| **MP12K** | 1 | 12000 | HiSeq2000 | Mate-Paired, 2×100 | 10.02 | 5.88 |
| **MP15K** | 1 | 16000 | HiSeq2000 | Mate-Paired, 2×100 | 2.89 | 1.25 |

**Table S3. Summary of genome sequencing for the female horse and the female mule.**

| Subject | Library type | Numbe of library | Insert size (bp) | Platform | Reads length (bp) |
|---|---|---|---|---|---|
| **Horse** | PE400 | 1 | 400 | Hiseq X-ten | Paired-End, 2×150 |
| **Mule** | PE400 | 1 | 400 | Hiseq X-ten | Paired-End, 2×150 |

**Table S4. Qualified data for the horse and mule genomes.**

| Sample | Raw data | | | | | | Qualified data | | | | | |
|---|---|---|---|---|---|---|---|---|---|---|---|---|
| | Reads Num. | Total Bases (bp) | N (%) | GC content | Q20 (%) | Q30 (%) | Reads Num. | Total Bases (bp) | N (%) | GC content | Q20 (%) | Q30 (%) |
| **Horse** | 698169770 | 104725465500 | 0.005 | 45 | 94.78 | 89.21 | 692398684 | 103775065988 | 0.006 | 45 | 95.08 | 89.61 |
| **Mule** | 766703794 | 115005569100 | 0.006 | 43.98 | 95.06 | 89.55 | 762403960 | 114360220780 | 0.006 | 43.97 | 95.25 | 89.8 |

**Table S5. Summary of mapping against the Thoroughbred horse reference genome (*EquCab3.0*).**

| Sample | Total reads | Mapped reads (%) | Total mapped bases (bp) | Avg. Depth (×) |
|---|---|---|---|---|
| **Donkey** | 860251582 | 843558716 (98.06%) | 97328930259 | 38.82× |
| **Horse** | 662435161 | 661240553 (99.82%) | 97441129059 | 38.87× |
| **Mule** | 669721430 | 666198582 (99.47%) | 97288042731 | 38.81× |

**Table S6. Sequencing depth coverage. Reads were aligned to the Thoroughbred horse reference genome (*EquCab3.0*) using BWA, and the frequency of each covered genome bases was calculated.**

| Sample | Coverage at least 1 × (%) | Coverage at least 10 × (%) | Coverage at least 20 × (%) |
|---|---|---|---|
| **Donkey** | 98.13 | 95.00 | 86.60 |
| **Horse** | 99.32 | 97.19 | 88.75 |
| **Mule** | 99.36 | 97.29 | 89.64 |

**Table S7. Genotype combinations for the Mendelian Inheritance.**

| Donkey genotype | Horse genotype | Mule genotype | Total SNPs num. |
|---|---|---|---|
| 0/0 | 0/0 | 0/0 | 0 |
| 0/0 | 0/1 | 0/0 | 577750 |
| 0/0 | 0/1 | 0/1 | 574537 |
| 0/0 | 1/1 | 0/1 | 237414 |
| 0/1 | 0/0 | 0/1 | 544963 |
| 0/1 | 0/0 | 0/0 | 523180 |
| 0/1 | 0/1 | 0/0 | 1853 |
| 0/1 | 0/1 | 0/1 | 4688 |
| 0/1 | 0/1 | 1/1 | 1394 |
| 0/1 | 1/1 | 1/1 | 1824 |
| 0/1 | 1/1 | 0/1 | 1887 |
| 1/1 | 0/0 | 0/1 | 10610326 |
| 1/1 | 0/1 | 0/1 | 262547 |
| 1/1 | 0/1 | 1/1 | 263257 |
| 1/1 | 1/1 | 1/1 | 549103 |

**Table S8. Genotype combinations for Mendelian inheritance Errors.**

| Donkey genotype | Horse genotype | Mule genotype | Total SNPs num. | Inherited state |
|---|---|---|---|---|
| 0/0 | 1/1 | 0/0 | 1134 | Inherited donkey |
| 0/1 | 0/0 | 1/1 | 666 | Inherited donkey |
| 0/1 | 1/1 | 0/0 | 24 | Inherited donkey |
| 1/1 | 0/0 | 1/1 | 9771 | Inherited donkey |
| 1/1 | 0/0 | 0/0 | 13178 | Inherited horse |
| 0/0 | 0/1 | 1/1 | 425 | Inherited horse |
| 1/1 | 0/1 | 0/0 | 287 | Inherited horse |
| 0/0 | 1/1 | 1/1 | 218 | Inherited horse |

**Table S9. Genotype combinations for *de novo* SNPs in the mule genome.**

| Donkey genotype | Horse genotype | Mule genotype | Total SNPs num. |
|---|---|---|---|
| 0/0 | 0/0 | 0/1 | 553 |
| 0/0 | 0/0 | 1/1 | 2 |
| 1/1 | 1/1 | 0/0 | 0 |
| 1/1 | 1/1 | 0/1 | 0 |
